# Suppression of urinary bladder urothelial carcinoma cell by the ethanol extract of pomegranate fruit through cell cycle arrest and apoptosis

**DOI:** 10.1186/1472-6882-13-364

**Published:** 2013-12-21

**Authors:** Song-Tay Lee, Min-Hua Lu, Lan-Hsiang Chien, Ting-Feng Wu, Li-Chien Huang, Gwo-Ing Liao

**Affiliations:** 1 Department of Biotechnology, Southern Taiwan University of Science and Technology, Nan-Tai Street, Tainan, Yung-Kung District 710, Taiwan; 2 Department of Life Sciences, University Road, National Cheng Kung University, Tainan 701, Taiwan

## Abstract

**Background:**

Pomegranate possesses many medicinal properties such as antioxidant, anti-inflammation and antitumor. It has been extensively used as a folk medicine by many cultures. Pomegranate fruit has been shown to have the inhibitory efficacy against prostate cancer and lung cancer *in vitro* and *in vivo*. It can be exploited in chemoprevention and chemotherapy of prostate cancer. In this study we examined the anti-cancer efficacy of pomegranate fruit grown in Taiwan against urinary bladder urothelial carcinoma (UBUC) and its mechanism of action.

**Methods:**

Edible portion of Taiwanese pomegranate was extracted using ethanol and the anti-cancer effectiveness of ethanol extract was evaluated by 3-(4,5-dimethylthiazol-2-yl)-2,5-diphenyltetrazolium bromide assay. Flow cytometry and western immunoblotting were exploited to uncover the molecular pathways underlying anti-UBUC activity of Taiwanese pomegranate ethanol extract.

**Results:**

This study demonstrated that Taiwanese pomegranate fruit ethanol extract (PEE) could effectively restrict the proliferation of UBUC T24 and J82 cells. Cell cycle analyses indicated that the S phase arrest induced by PEE treatment might be caused by an increase in cyclin A protein level and a decrease in the expression of cyclin-dependent kinase 1. The results of western immunoblotting demonstrated that PEE treatment could not only evoke the activation of pro-caspase-3, -8,-9 but also increase Bax/Bcl-2 ratio in T24 cells. The above observations implicated that PEE administration might trigger the apoptosis in T24 cells through death receptor signaling and mitochondrial damage pathway. Besides we found that PEE exposure to T24 cells could provoke intensive activation of procaspase-12 and enhance the expressions of CHOP and Bip, endoplasmic reticulum (ER) stress marker, suggesting that ER stress might be the cardinal apoptotic mechanism of PEE-induced inhibition of bladder cancer cell.

**Conclusions:**

The analytical results of this study help to provide insight into the molecular mechanism of induced bladder cancer cell apoptosis by pomegranate and to develop novel mechanism-based chemopreventive strategy for bladder cancer.

## Background

Many epidemiologic and scientific evidences strongly imply that plant-derived phytochemicals may provide a critical clue for cancer prevention or treatment [1-5]. Pomegranate (*Punica granatum*, Punicaceae) has been extensively used as a folk medicine in many countries [6]. Edible portions (80% of total fruit weight) of pomegranate fruit comprise 80% juice and 20% seed with nutritious ingredients of crude fibers, pectin, sugars, and polyphenols [tannins (mainly ellagitannins), flavonoids and anthocyanins] which confer the fruit with potent antioxidant activity [7-10].

Cumulative findings have demonstrated that pomegranate fruit have various anti-tumor capabilities. Anthocyanin/ellagitannin-rich pomegranate fruit extract (PFE) prepared from pomegranate edible portion with 70% acetone was found to evoke the apoptotic impacts on human lung cancer A549 cells by hampering NF-κB as well as MAP kinase pathway (p38, PI3K/Akt, JNK and Erk) and the down-regulation of cell cycle-regulatory proteins functioning in the G_1_ stage [11]. In primary lung tumor animal model, PFE also inhibits tumor growth/progression/angiogenesis by the suppression of NF-κB, MAP kinase pathway and mTOR signaling pathway [12]. In addition to the influences on lung cancer, various preparations of pomegranate were investigated on human prostate cancer. Oral feeding of 0.1% and 0.2% (w/v) PFE significantly restricts the tumor growth of androgen-responsive CWR22R*v1* cell in athymic nude mice. PFE can cause G_1_-phase arrest of prostate cancer PC3 cell through dwindling the expressions of cyclins D and E and increasing the levels of cyclin-dependent kinase-2, -4 and -6, and thus resulting in an increase in WAF1/p21 as well as KIP1/p27 [13]. It also provokes PC3 cell apoptosis by over-expression of proapoptotic Bax and Bak while down-regulating anti-apoptotic Bcl-2 as well as Bcl-XL. Other investigations on prostate cancer found that pomegranate polyphenols, ellagitannin-rich extract (PE) made from fruit skins standardized to ellagitannins, can restraint prostate cancer cell caused by chronic inflammation via suppressing NF-κB pathway, which is well-established signaling pathway mediating the inflammation relevant to cancer [14]. PE was also found to thwart angiogenesis in prostate cancer through down-regulation of hypoxia-inducible factor 1-α (HIF-1α) which takes part in the expression of vascular endothelial growth factor (VEGF) by transcriptional regulation [15]. A phase II clinical study for men with rising PSA after surgery or radiotherapy demonstrated that pomegranate juice can statistically prolong PSA doubling time, suggesting potential preventive efficacy of pomegranate in human prostate cancer [16].

Bladder cancer is the most prevalent tumor of urinary tract worldwide. Among the broad range of histological heterogeneous tumor types that derive from the urothelium lining of urinary bladder and ureters, urothelial carcinoma is the most common which constitutes more than 90% of bladder cancer cases in developed countries [17]. Although the majority of urinary bladder urothelial carcinoma (UBUC) is papillary and non- or superficially invasive, cured most of the time by curettage, some UBUCs develop relentless local recurrence followed by lethal distal spreading [18,19]. Thus pomegranate may be a potential chemopreventive source against UBUC development and recurrence based upon the aforementioned evidences. Nevertheless nowadays there is no literature showing that pomegranate foils UBUC.

In this study we examine the inhibitory function of pomegranate fruit ethanol extract (PEE) in UBUC. We found that PEE could retard UBUC T24 and J82 cell proliferation. The inhibitory effects might be attributed to S-phase arrest provoked by PEE via de-regulating the cylin A and cdc2 or cell apoptosis within T24 cell. Our results showed that PEE could evoke T24 cell apoptosis via mitochondrial pathway, death receptor pathway and endothelium reticulum (ER) stress. Nevertheless, stronger ER stress response was observed in T24 cell. Furthermore PEE-evoked ER stress might dys-regulate vasolin-containing protein (VCP) to activate pro-caspase-12, and thus induce the apoptosis in T24 cell.

## Methods

### Collection and identification of plant materials

The fruits of *P. granatum* were field collected from a farm land (22°41'59.3267” N, 120°30'45.1836” E) located in a small township Jiuru, Pingtung county, southern Taiwan from August to September, 2012. The plant specimens were identified by Liao, G.-I. and pressed/dried for voucher specimens (Nan-Kai Lin, STUSTG308-001 to STUSTG308-003) deposited in the herbarium of Taiwan forestry research Institute (TAIF), Taiwan.

### Preparation of pomegranate fruit ethanol extract (PEE)

Fresh pomegranate fruit was peeled and the edible portion was squeezed with gauze. The subsequent juice was concentrated by freeze dried with 37.5 ml juice to produce 4.13 g of powder. The powder was first extracted with ethylacetate (EtOAc) at a ratio of 1:3 (w/v) in 50 mL-poly-propylene (PP) centrifugation tube with 360° rotation for 16 hours at room temperature. After extraction, the residue was collected with centrifugation at 10,000 × g and the supernatant was vacuum dried. After centrifugation, the residue was extracted with 70% ethanol as described in EtOAc extraction. After extraction, 17 mg [yield 0.41% (w/w)] and 2.96 g [yield 71.7% (w/w)] of the products were obtained respectively from EtOAc and EtOH extraction of 37.5 ml juice.

### Cell lines

Human urinary bladder urothelial carcinoma (UBUC) T24 cell, which is recognized as high grade and invasive, was purchased from Bioresource Collection and Research Center, Hsinchu, Taiwan and cultured at 37°C in McCoy's5A [GIBCO (Life technologies), Grand Island, N.Y., U.S.A.], supplemented with 10% (v/v) fetal bovine serum (FBS). Human papillomavirus E7 immortalized uroepithelial cell was kindly provided by Professor Hsiao-Sheng Liu from department of microbiology and immunology, college of medicine, National Cheng Kung University, Tainan, Taiwan and maintained as described previously [20]. UBUC J82 cells recognized as high grade was offered by Dr. Chien-Feng Li from Department of Pathology, Chi-Mei Medical Center, Tainan, Taiwan and maintained at 37°C in Dulbecco's Modified Eagle Medium supplemented with 10% (v/v) FBS (GIBCO, Grand Island, N.Y., U.S.A.).

### 3-(4,5-dimethylthiazol-2-yl)-2,5-diphenyltetrazolium bromide (MTT) assay

Appropriate concentrations of PEE were added to a 96-well plate already seeded with 5,000 human T24 cells, 6,000 human J82 cells or 3,000 human E7 cells per well. After exposure for the indicated time duration, 20 μl of MTT solution (Merck, Damstadt, German) (5 mg/ml PBS) was added to each well and the plate was incubated at 37°C for 4 hours. After medium removal, 200 μl of DMSO was added to each well and the plate was gently shaken for 5 minutes. The absorbance was determined at 540 nm. Quadruplicate wells were applied to each concentration for a specific time period. 0.002% (v/v) DMSO (vehicle)-treated bladder cancer cells were employed as the control.

### Cell cycle analysis of PEE-treated human T24 cells

Human T24 cells were plated onto a 6-well plate at 8 × 10^5^/well and cultivated overnight. Then 50 or 100 μg/ml of PEE was added onto each well and the cells were harvested with trypsinization at the indicated time period. The harvested cells were incubated with 500 μl of ice-cold 70% ethanol/well at 4°C overnight. After ethanol treatment, the cells were washed with 1 ml ice-cold PBS/well, re-suspended in 100 μl PBS/well and incubated with 300 μl propidium iodide (PI) (Sigma, Saint Louis, Missouri, USA) solution (3 μl RNase and 20 μg PI per ml)/well in the dark at 37°C for 30 minutes. The stained cells are analyzed by FACSCalibur flow cytometer (Becton Dickinson). 0.002% (v/v) DMSO-treated human T24 cells were used as the control and analyzed as described above.

### Annexin V/PI staining of PEE-exposed human T24 cells

Human T24 cells were plated onto a 6-well plate at 4 × 10^5^/well and cultivated overnight. Then 50 or 100 μg/ml of PEE was added onto each well and the cells were harvested with trypsinization at the indicated time period. The harvested cells were washed with 1 ml ice-cold PBS/well and re-suspended in 100 μl binding buffer/well. Then 2 μl of annexin V conjugated with FITC (Strong Biotech Corporation, Tainan, Taiwan) plus 2 μl of PI solution (Sigma, Saint Louis, Missouri, USA) were mixed with the re-suspended cells and incubated in the dark on ice for 15 minutes. After staining, the cells were analyzed using FACSCalibur, Becton Dickinson. 0.002% (v/v) DMSO-treated human T24 cells were utilized as the control and analyzed as described above. As to annexin V/PI experiments incorporated with caspase-3 Z-DEVD-FMK inhibitor (BD Bioscience), human T24 cells were incubated with 2 μM inhibitor for 4 hours. Then inhibitor-pre-incubated human T24 cells were exposed in the presence or absence of 100 μg/ml PEE for 48 hours. Apoptosis was detected as described above.

### Western immunoblotting

After appropriate treatment with PEE, human T24 or J82 cells were harvested and lysed in lysis buffer [10 mM Tris (pH 8.0), 0.32 M sucrose, 1% (v/v) Triton X-100, 5 mM EDTA, 2 mM DTT, and 1 mM PMSF]. After determining its protein concentration using Bio-Rad DC protein assay kit, equal volume of 2× sample buffer [0.1 M Tris (pH 6.8), 2% SDS, 0.2% β-mercaptoethanol, 10% (v/v) glycerol, and 0.0016% (w/v) bromophenol blue] was combined with the cytoplasmic extract. Appropriate amounts of the lysates were separated by electrophoresis at 100 V with 10% (w/v) sodium dodecyl sulfate (SDS)-polyacrylamide gels, and further transferred on to a PVDF membrane (Strategene, La Jolla, CA, USA). After blocking for 1 hour in 3% (w/v) bovine albumin serum (BSA) at room temperature, membranes were hybridized overnight at 4°C with primary antibodies. The primary antibodies used in this study were listed in supplementary document. The membranes were washed and probed with suitable secondary antibodies for 1 hour at room temperature. Secondary antibodies binding on the membrane were detected by chemiluminescence ECL detection system (Amersham-Pharmacia Biotech Inc., Piscataway, NJ, USA) using Fujifilm LAS-3000 Luminescent Image Analyzer (Fujifilm Corporation, Tokyo, Japan). The intensity of each protein band was quantified by PDQUEST Quantity One software (Bio-Rad Laboratory, Hercules, CA) and normalized with actin protein expression level.

### Statistical analyses

Student’s *t*-test was used to assess the significance between controls and treatments. Statistical analysis was performed using STATISTICA Advanced V10.0 (Statsofa Holdings, Inc., Tulsa, Oklahoma, USA).

## Results

### The suppressive influences of PEE on UBUC cell proliferation

**Figure 1:**
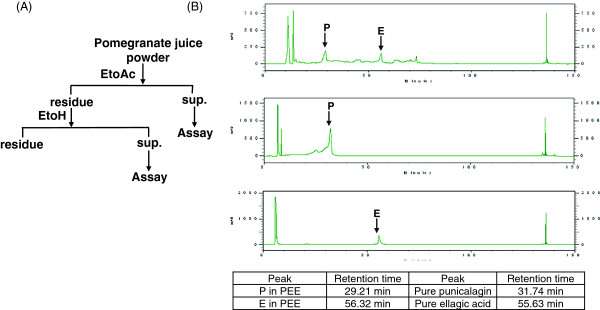
**The extraction procedure of PEE and HPLC results.** HPLC was underwent as described in Methods. **(A)** Local pomegranate juice was extracted sequentially by ethylacetate and 70% ethanol (supernatant, abbreviated Sup.). **(B)** HPLC profiles of PEE. The profiles of pure unicalagin and pure ellagic acid were indicated above the panels respectively. The possible punicalagin and ellagic acid peaks in PEE were shown by an arrow and a letter.

**Figure 2:**
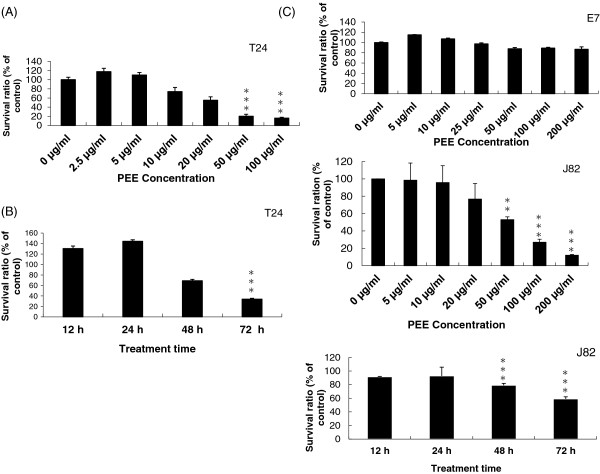
**The inhibitory efficacy of PEE on UBUC cells. (A)** Human T24 or J82 cells were incubated with increasing concentration of PEE for 72 h and cell viability was measured by MTT assay as described in Methods. **(B)** Time-dependent response experiments were underwent by treating human T24 or J82 cells with 50 μg/ml of PEE for various time durations. **(C)** The selectivity of PEE was determined by dose-response against E7 cell carried out by exposing the cells in the presence of the indicated concentrations for 72 h. All the diagrams in Figure 2 were the typical result of three independent experiments. All the data were expressed as mean ± standard deviation (S.D.) of the mean of four wells performed in triplicate. ** and *** denoted *P* < 0.01 and *P* < 0.001 respectively as compared to untreated cell using Student’s *t*-test.

In this work, fresh pomegranate juice was sequentially extracted using solvent with increasing polarity as shown in Figure 1A. There are many well-documented literatures demonstrating that pomegranate possesses anti-cancer effectiveness against various tumors. Thus we speculated that pomegranate might inhibit UBUC. To evaluate our speculation, PEE was evaluated on T24 and J82 cells for its inhibitory influences on cell proliferation. T24 or J82 cells were incubated for 72 hours with or without various concentrations of PEE (5 to 200 μg/ml). The experimental results in Figure 2 demonstrated that the influence of PEE could be observed in T24 cells after treatment for 72 hours with 10 μg/ml and in J82 cells for 72 hours with 50 μg/ml. The effectiveness was more obvious in T24 cells than that in J82 cells. However, the suppressive effects of PEE were more prominent after treatment for 72 hours with >50 μg/ml both in T24 and J82 cells. After 72 hours, approximately 80% of T24 or J82 cells were dead after incubation with 100 μg/ml of PEE. Besides, 50 μg/ml PFE- exposure to T24 cell for various periods (0 to 72 hours) inhibited cell proliferation. The observation results suggested that PEE could affect the viability of UBUC cells.

The inhibitory influence of PEE on cell survival observed in T24 or J82 cells suggested that PEE might similarly impact normal cells. E7 cells were employed to examine this likelihood. As indicated in Figure 2C, almost all the E7cells survived after 72 hours treatment in all the PEE concentrations tested. However, PEE could impede bladder cancer T24 or J82 cell in a dose-dependent manner. Thus normal and cancer cell lines exhibited differential sensitivity to PEE.

In order to get insight into the constituents present in PEE, PEE was subjected to HPLC and the results were shown in Figure 1. HPLC results demonstrated that most compounds in PEE were present in the medium polar area (retention time from 40 to 90 minutes). A possible ellagic acid peak and a potential punicalagin peak appeared in HPLC profile of PEE when compared to those of pure ellagic acid and pure punicalagin in Figure 1. These two compounds seem to be the major ingredients in PEE. Consistent with HPLC results, GC/MS results showed that furanaldehyde, furanketone and pryrrolo[1,2-α]pyrazine compounds were present in PEE (Table 1). However, further investigation was required to decide whether these compounds were linked to the anti-cancer activities of PEE.

**Table 1:** The composition of PEE evaluated by GC/MS

**Constituents**	**Peak area (%)**^**a**^
Furfural	5.34
2-methyl-2-Hexanol	0.74
5-methyl-2-Furancarboxaldehyde	1.02
Phenylacetaldehyde	0.96
Furyl hydroxymethyl ketone	1.03
3,5-dihydroxy-2-methyl-4H-Pyran-4-one	0.80
Hydroxy methyl furfural	16.85
3-nitro-Benzenemethanol	3.08
Hexahydro-3-(2-methylpropyl)-pyrrolo[1,2-α]pyrazine-1, 4-dione	2.13
Hexahydro-3-(phenylmethyl)-pyrrolo[1,2-α] pyrazine-1, 4-dione	3.13
Diethyl phthalate	0.78
Total	35.86

^a^Only the compounds with identification score (SI) > 90 were listed.

### PEE interfered with human T24 cell proliferation by inducing cell cycle arrest

**Figure 3:**
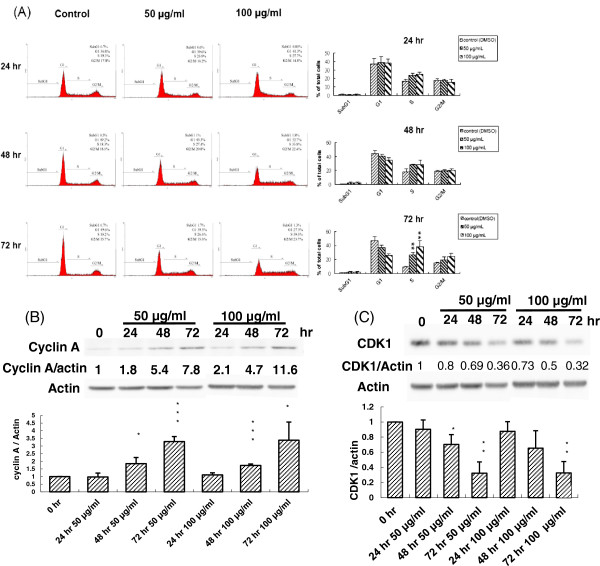
**Induction of human T24 cell cycle arrest by PEE.** Human T24 cells were incubated with 50 or 100 μg/ml of PEE for the indicated time periods and cell cycle analyses were determined by flow cytometry as described in section 2. The representative diagram was shown in **(A)**, and the mean ± S.D. of three independent experiments was indicated in the right panel. Human T24 cells were treated with 50 or 100 μg/ml of PEE for different time durations and cyclin A as well as CDK1 in cell lysates were detected by western blotting with the antibodies against cyclin A **(B)** and CDK1 **(C)** respectively as described in section 2. The blot in each diagram was the representative result of three independent experiments and the densitometer-intensity data (mean ± S.D.) were shown under each blot. *, ** and *** represented P < 0.05, P < 0.01 and P < 0.001 respectively as compared to untreated cell using Student’s *t*-test.

According to the above results, PEE could invoke an inhibitory impact on human T24 cell proliferation. To discover which mechanism was involved in the inhibition of human T24 cell proliferation, PEE-exposed human T24 cells were first investigated with cell cycle analysis using flow cytometry. Cell cycle analyses conducted after incubation of human T24 cell with a series of increasing concentration for various durations demonstrated that PEE arrested human T24 cells in S phase in a dose-and-time dependent response (Figure 3A). After exposure to 100 μg/ml for 72 hours, 39.5% of the cells were arrested at the S phase, whereas only 23.7% were at the G_2_/M phase and 27.3% at the G_1_ phase of the cell cycle. Previous evidences reported that during normal cell cycle cyclin A- CDK1 (cyclin-dependent kinase 1) complex begins to form in late S phase and is over-expressed in G_2_ phase to result in the progression from S phase to G_2_/M phase [21,22]. Thus western immunoblotting was carried out to detect the impact of PEE on the expressions of cyclin A and CDK1 in human T24 cells. The subsequent findings showed that cyclin A level was increased whereas CDK1 level was decreased in PEE-treated human T24 cell (Figure 3), suggesting that PEE evoked S phase arrest in human T24 cell possibly through the dys-regulation of cyclin A and CDK1.

In addition, PEE treatment increased G_2_/M population as compared to those of the untreated T24 cells. However, G_2_/M arrest induced by PEE was not statistically significant (Additional file 1: Table S1). Western immunoblotting indicated that PEE exposure resulted in the over-expressions of CDC25C, pCDC25 and cyclin B1 in T24 cells (Additional file 2: Figure S1) instead of under-expression. Thus we anticipated that PEE could not cause G_2_/M arrest.

### PEE dampened human T24 cell proliferation by evoking the apoptosis via mitochondrial pathway as well as death receptor signaling

**Figure 4:**
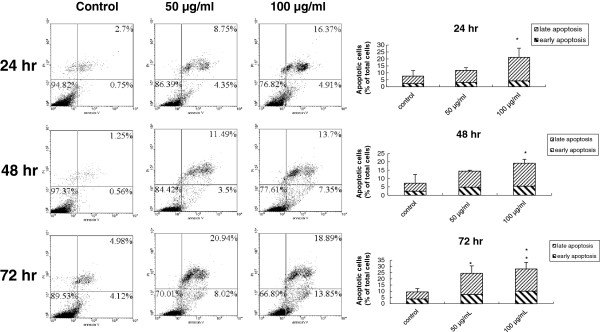
**PEE-provoked human T24 cell apoptosis.** Apoptotic effect was observed by treating human T24 cells with 50 or 100 μg/ml of PEE for the indicated time periods and the effect was measured by the annexin V/PI staining as described in section 2. The representative data was posted in **(A)** and the mean ± S.D. of three independent experiments was demonstrated in **(B)**.

In addition to cell cycle arrest, we also explored the likelihood that the inhibitory effects of PEE on human T24 cell proliferation might be attributed to the apoptosis. The data of cell cycle analyses indicated that PEE exposure could induce the formation of hypodiploid subG_1_ peak in a dose-and-time dependent response as compared to those of the control, implicating that PEE could cause the DNA fragmentation in human T24 cells, although the percentage of subG_1_ population in the cell profile was low (< 2.3%). The results of annexin V/PI staining indicated that after incubation with PEE in a dose-and-time dependent manner it could provoke phosphatidylserine (PS) translocation in the cell membrane, which is an initial sign of apoptosis that leads to the subsequent membrane leakage (late apoptotic cell) and thus increases the percentage of early and late apoptotic cells (Figure 4).

**Figure 5:**
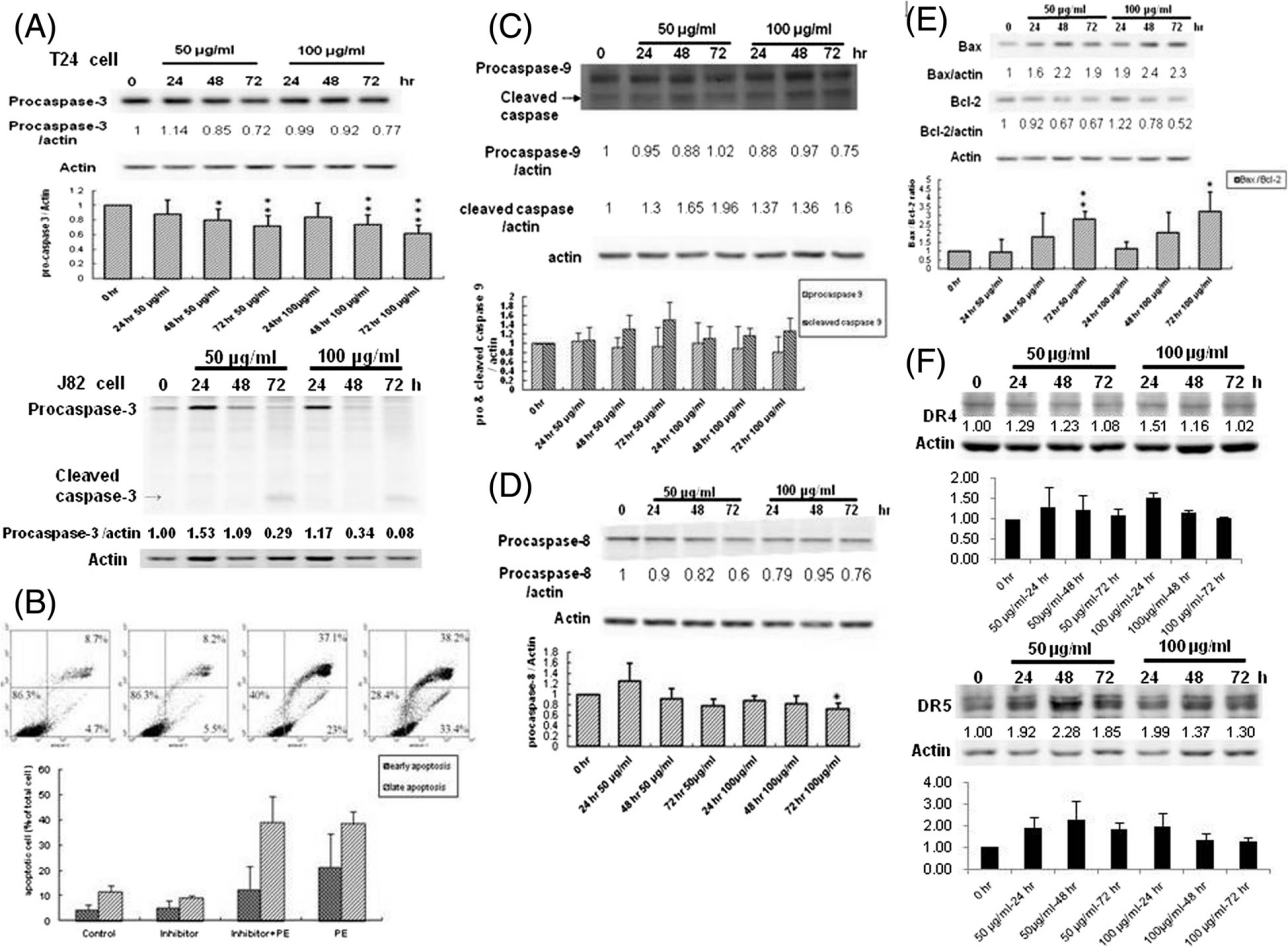
**PEE-evoked human T24 cell apoptosis via the mitochondrial pathway and death receptor pathway.** Human T24 or J82 cells were treated with 50 or 100 μg/ml of PEE for different time durations. Various effector proteins involved in apoptosis were detected by western blotting with the antibodies against procaspase-3 **(A)**, procaspase-9 **(C)**, procaspase-8 **(D)**, Bax/BcL-2 **(E)**, DR4 and DR5 **(F)** respectively as described in section 2. T24 cells were investigated in all panels except panel **A** as indicated. The blot in each diagram was the typical result of three independent experiments except pro-caspase-3 examination in J82 cell (one experiment) and DR4 as well as DR5 detection (two independent experiments) in T24 cells. The densitometer-intensity data (mean ± S.D.) were shown under each blot. **(B)** For the counteract study, human T24 cells were incubated with 2 μM of caspase-3 inhibitor Z-DEVD-FMK for 4 hours and then 100 μg/ml of PEE was incorporated with the inhibitor-pretreated cells for 48 hours. After incubation, the cells were collected for annexin V/PI analyses.

Although the aforementioned results showed that PEE might induce the apoptosis in human T24 cell, cytometric analysis cannot decipher whether PEE results in the apoptosis by death receptor signaling, mitochondrial damage pathway or ER stress. In order to define the molecular mechanism by which PEE invoked the apoptosis, western immunoblotting was conducted to examine the processing and activation of caspase-3 which is considered to play a central role in both mitochondrial damage and death receptor signaling, and can be activated by caspase-8 in death receptor signaling and by caspase-9 in mitochondrial damage. As shown in Figure 5A, the amount of pro-caspase-3 decreased in a time-dependent response, implicating that caspase-3 was activated and the apoptosis might be triggered by mitochondrial damage or death receptor signaling. To reinforce the observation of PEE-induced apoptosis via activation of pro-caspase-3, PEE-administrated T24 cell was incubated with caspase-3 inhibitor Z-DEVD-FMK. Significantly reduced numbers of early apoptotic T24 cells present in inhibitor/PEE treatment of annexin V/PI staining experiments showed that Z-DEVD-FMK could antagonize the apoptotic effects of PEE on human T24 cell as compared to those of sole PEE exposure (Figure 5B). Similarly pro-caspase-3 was activated in PEE-treated J82 cell in a dose-and-time relied response (Figure 5A).

To further uncover the apoptotic mechanism mediated by PEE, the activation of caspase-8 and -9 in PEE-exposed human T24 cells were examined by western immunoblotting. The results in Figure 5C and 5D demonstrated that the levels of pro-caspase-9 and -8 dwindled temporally in 50 μg/ml and 100 μg/ml PEE-administrated human T24 cells whereas activated caspase-9 amount increased, implying that PEE-induced human T24 cell apoptosis might be attributed to the mitochondrial damage pathway and death receptor signaling. Backing up the involvement of mitochondrial damage pathway in PEE-provoked apoptosis, further experimental results found that Bcl-2 level decreased while Bax amount increased (Figure 5E). To confirm death receptor pathway was activated in PEE-treated T24 cell, western immunblotting with DR4 and DR5 antibodies was conducted and showed that DR4 and DR5 was up-regulated in PEE-incubated T24 cells, implying that death receptor was involved in the apoptosis induced by PEE in T24 cells (Figure 5F).

### PEE caused endoplasmic reticulum (ER) stress to induce the apoptosis in T24 cell

**Figure 6:**
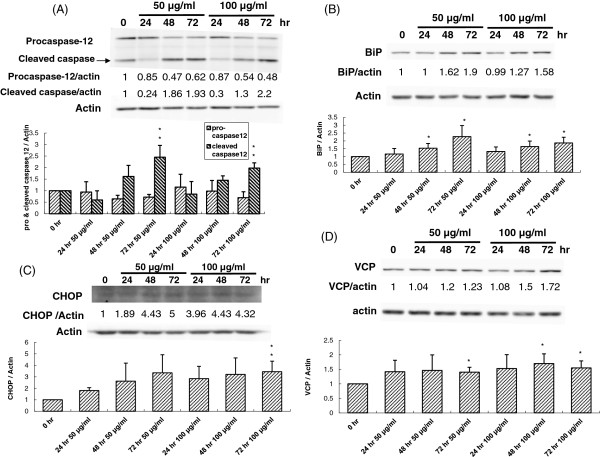
**PEE-evoked ER stress in human T24 cell.** Human T24 cells were treated with 50 or 100 μg/ml of PEE for different time durations. Various effector proteins participating in ER stress were detected by western blotting with the antibodies against procaspase-12 **(A)**, CHOP **(B)**, BiP **(C)** and VCP **(D)** respectively as described in section 2. The blot in each diagram was the representative result of three independent experiments and the densitometer-intensity data (mean ± S.D.) were shown under each blot.

The above findings demonstrated that PEE caused human T24 cell apoptosis possibly via mitochondrial damage and death receptor signaling. However, it is likely that PEE exposure could also result in ER stress, and thus cell apoptosis in human T24 cell. Hence the processing and activation of pro-caspase-12, which is regarded as an essential effector in ER stress, was examined by western immunoblotting. The follow-up discovery showed that pro-caspase-12 level was reduced while cleaved pro-caspase-12 (activated form) amount increased in PEE-exposed T24 cells in a temporal response (Figure 6A), implicating that PEE might provoke an ER stress in human T24 cell. To further clarify the impacts of PEE on ER stress, western immunoblotting was conducted to detect CHOP and Bip, the ER stress markers whose over-expression will initiate ER stress. The follow-up results illustrated that both CHOP and Bip levels were increased in PEE-treated human T24 cells (Figure 6B and 6C), reinforcing that PEE treatment caused ER stress in human T24 cell. Previous findings implicated that during chemically induced ER stress, valosin-containing protein (VCP) triggers processing and activation of pro-caspase-9 and -12 [23]. This investigation found that PEE exposure augmented the VCP expression in T24 cells using western immunoblotting (Figure 6D), implying that PEE treatment might evoke ER stress in T24 cells through VCP de-regulation.

## Discussion

Pomegranate has been illustrated to possess anti-carcinogenic properties that may suppress various cancers. Many research applications have been made to determine the chemopreventive effects of pomegranate on lung cancer and prostate cancer but not regarding bladder cancer. They have shown that pomegranate polyphenols can inhibit prostate cancer and lung cancer both in vitro and in vivo [10-15]. Above that, following primary treatment, with daily consumption of 8 ounces of pomegranate fruit juice, prostate cancer patients can extensively lengthen prostate-specific antigen doubling time [16]. Thus pomegranate may be a potential chemopreventive source against UBUC progression and recurrence based upon previous evidences. The incidence of bladder cancer in southwestern coast of Taiwan, where blackfoot disease is endemic, is unusually high. Epidemiological studies have established a link between arsenic and bladder cancer [24]. Since there is no eveidence-based literature showing the inhibitory efficacy of pomegranate to UBUC, and high incidence as well as frequent recurrence of UBUC still prevails in southwestern Taiwan, this study was intended to examine if pomegranate could impede UBUC growth. The results of this study hope to provide the basis for future development in adopting local pomegranate as a chemopreventive source against UBUC in Taiwan.

In this study we found that PEE treatment could inhibit UBUC T24 and J82 cell proliferation in a time-and-dose dependent response. Cell cycle analyses demonstrated that PEE exposure caused S-phase arrest in human T24 cells and de-regulation of cyclin A as well as CDK1, the complex of which is essential for S to G_2_/M transition. Similar results were reported by Shi et al. (2003) [25] that the treatment of prostate cancer cell with BBSKE, a novel organoselenium compound, results in S-phase arrest and increase in the protein levels of cyclin A, cyclin E, and p21, but decrease in the levels of cyclin B1, cyclin D1, and CDK4 [25]. Furthermore, BBSKE augments Bax/Bcl-2 ratio, and thus induces apoptosis. In contrast, PFE (acetone extract of pomegranate juice) incubation results in G_1_ phase arrest in prostate cancer and lung cancer cells [11-13].

Besides cell cycle arrest, the inhibitory activity of PEE toward UBUC cells might be attributed to apoptosis. MTT results demonstrated that PEE exhibited the stronger inhibitory effect on T24 cell than J82 cell. Moreover, both of T24 and J82 cells belong to high grade UBUC cells. Thus we chose T24 cells for further exploration of the molecular pathway involved in apoptosis. The results of annexin V/PI staining demonstrated that PEE treatment could evoke T24 cell apoptosis in a dose-and-time dependent manner. The investigation of processing and activation of various proapoptotic caspases revealed that PEE treatment activated pro-capspase-3, -8 and -9 in T24 cells, implying that mitochondrial pathway and death receptor pathway might be involved in initiating apoptosis. Furthermore we found that pro-caspase-12 level significantly reduced while the amount of activated caspase-12 prominently increased in PEE-exposed T24 cells in a temporal response as compared to those of pro-caspase-3, -8 and -9, indicating that ER stress might be the dominant pathway to evoke apoptosis although mitochondrial pathway and death receptor pathway also could induce apoptosis in T24 cells. Over-expression of ER stress markers, CHOP and BiP, further backed up the observation of PEE-induced ER stress. Previous reports demonstrated that when ER stress was induced by chemicals, VCP might become a key component of a large ER-associated complex which triggered processing and activation of pro-caspase-9 and -12 [23]. The aforementioned phenomenon implicated that PEE exposure might provoke ER stress in human T24 cells followed by VCP over-expression to trigger the activation of pro-caspase-9 and -12. Besides, pro-caspase-3 was activated in a dose-and-time dependent manner in PEE-treated J82 cell, indicating that similar apoptotic mechanism might be provoked by PEE exposure.

In resemblance to our findings, the examination of PFE (acetone extract) reported by Malik et al. (2005) found that PFE treatment to prostate cancer and lung cancer cells could induce G_1_ phase arrest and provoke prostate cancer cell apoptosis by dys-regulation of both cyclins and CDKs and down-regulation of anti-apoptotic Bcl-XL and Bcl-2 [13]. However, few evidences supported that PFE could induce cell apoptosis in prostate cancer and lung cancer cells. This study provided extensive findings which showed that PEE administration could evoke UBUC cell apoptosis.

## Conclusions

In conclusion, PEE treatment could suppress UBUC cell and might trigger the apoptosis in bladder cancer cell through death receptor signaling and mitochondrial damage pathway. More importantly PEE exposure could provoke intensive activation of ER stress which might be the cardinal apoptotic mechanism of PEE-induced inhibition of bladder cancer cell. The analytical results of this study help to provide insight into the molecular mechanism of inducing bladder cancer cell apoptosis by pomegranate and to develop novel mechanism-based chemopreventive strategy for bladder cancer.

## Competing interests

The authors declare no financial or commercial conflict of interest.

## Authors’ contributions

MHL and LHC performed most of western immunoblotting experiments. LCH carried out most of apoptosis assays. GIL identified pomegranate and deposited the plant. STL participated in the project design, contributed to the data interpretation and wrote the initial drafts of the manuscript. TFW carried out the preparation of PFE and contributed to the conception and design of the entire study, the data interpretation, the initial draft and final editing of the manuscript. All authors have read and approved the manuscript for publication.

## Pre-publication history

The pre-publication history for this paper can be accessed here:

http://www.biomedcentral.com/1472-6882/13/364/prepub

## Supplementary Material

Additional file 1 Supplementary methods, supplementary tables and supplementary figure legends. Click here for file

Additional file 2 Supplementary figures. Click here for file
