# Supplementary material for: Suppression of urinary bladder urothelial carcinoma cell by the ethanol extract of pomegranate fruit through cell cycle arrest and apoptosis
Source: BMC Complement Altern Med. 2013 Dec 21;13:364. doi: 10.1186/1472-6882-13-364 (PMC3878077; doi:10.1186/1472-6882-13-364)
Supplement: Additional file 1 — Supplementary methods, supplementary tables and supplementary figure legends. [file 1472-6882-13-364-S1.docx]

**Supplementary methods**

**Analysis of PEE by gas chromatography/mass spectrometry (GC/MS)**

The chemical composition of PEE was analyzed by GC/MS using a SHIMADZU QP2010 GC/MS with a DB-5 column (30m, film0.25 um ,ID 0.25 mm). The temperature of the column was programmed from 45 ºC to 270 ºC at 5 ºC/min and the injector or detector temperature for the analysis was about 250 ºC. Helium was used as the carrier gas at a flow rate of 0.82 ml/min. The mass spectrometer was operated in electron-impact ionization (EI) mode with 70 eV energy. The identification of the chemical constituents was based on matching their recorded mass spectra with those obtained from the FFNSC1.2, NIST05 and NIST05s library spectra provided by the software of GC/MS system.

**Separation of PEE by high performance liquid chromatography (HPLC)**

The HPLC system consisted of a Hitachi liquid chromatograph equipped with one pump (Hitachi L-7100), an auto sampler injector (Hitachi L-7200) and a UV-VIS detector (Hitachi L-7420). The chromatographic separation was performed with Thermo Hypersil^®^ BDS C18 column (250 mm x 4.6 mm, 5 μm) to dissect the ethanol extract. The extract was eluted at the flow rate of 0.6 ml/min for 0 to 130 minutes using the 0.1 % (v/v) TFA/acetonitrile gradient system in which the acetonitrile percentage was increased 5 % (v/v) every 10 minute and 1 ml/min for 131 to 150 minutes with 100 % (v/v) acetonitrile. 10 mg/ml of PEE was made up with 100 % methanol while 1 mg/ml of pure purnicalagin (Sigma, Saint Louis, MO, USA) and 1 mg/ml of pure ellagic acid (Alfa Aesar, heysham, England) was made up with 100 % methanol and DMSO respectively.

**Primary antibody**

pro-caspase-3 (BD Biosciences, San Diego, CA, USA, 1:1000), pro-caspase-8 (GeneTex, Irvine, CA, USA, 1:200), pro-caspase-9 (GeneTex, Irvine, CA, USA, 1:500), pro-caspase -12 (Rockland, Gilbertsville, PA, USA, 1:500), CHOP(Spring Bioscience, Pleasanton, CA, USA, 1:500), Bip (BD Biosciences, San Diego, CA, USA, 1:1000), Bcl-2 (Santa Cruz, Dallas, Texas, USA, 1:200), Bax (Santa Cruz, Dallas, Texas, USA, 1:200), cyclin A (Bioworld, Louis Park, MN, USA, 1:500), cdc2 (Spring Bioscience, Pleasanton, CA, USA, 1:500), valosin containing protein (Abnova, Walnut, CA, USA, 1:2000), DR4 (Gene Tex, Irvine, CA, USA, 1:1000) DR5 (Gene Tex, Irvine, CA, USA, 1:2000), p-CDC25C (Cell signaling, Danvers, MA,USA, 1:1000), CDC25C (Gene Tex, Irvine, CA, USA, 1:2000), cyclin B1 (Gene Tex, Irvine, CA, USA, 1:2000)

**Supplementary figure legends**

**Figure S1 The expressions of CDC25C, pCDC25C and cyclin B1.** Human T24 cells were treated with 50 or 100 μg/ml of PEE for different time durations. Various effector proteins involved in apoptosis were detected by western blotting with the antibodies against pCDC25C (A), CDC25C (B) and cyclin B1 (C).

**Table S1** The percentage of cell cycle population.

| **Time** | **PEE** | **sub G1 (%)** | **G1 (%)** | **S (%)** | **G2/M (%)** |
| --- | --- | --- | --- | --- | --- |
|  | **control** | 1.13 ± 0.75 | 36.87 ± 6.55 | 16.83 ± 2.36 | 17.97 ± 1.56 |
| **24 h** | **50μg/ ml** | 0.67 ± 0.4 | 38.63 ± 7.4 | 23.77 ± 2.58^＊^ | 17.63 ± 1.56 |
|  | **100μg/ ml** | 0.98 ± 0.76 | 38.37 ± 4.82 | 24.8 ± 2.72^＊^ | 15.23 ± 4.07 |
|  | **control** | 0.57 ± 0.31 | 44.1 ± 4.45 | 18.37 ± 3.9 | 19.1 ± 0.87 |
| **48 h** | **50μg/ ml** | 2.23 ± 1.37 | 40.2 ± 3.26 | 27.83 ± 1.02^＊^ | 19.43 ± 1.4 |
|  | **100μg/ ml** | 2.53 ± 1.36 | 34.6 ± 3.91^＊^ | 28.13 ± 6.96 | 20.03 ± 2.45 |
|  | **control** | 1.07 ± 0.4 | 47.17 ± 5.38 | 9.37 ± 0.8 | 15.23 ± 0.45 |
| **72 h** | **50μg/ ml** | 2.17 ± 0.57 | 36.9 ± 3.44^＊^ | 26.73 ± 3.8^＊＊^ | 19.53 ± 4.11 |
|  | **100μg/ ml** | 1.9 ± 0.53 | 25.93 ± 2.28^＊＊^ | 38.47 ± 8.3^＊＊^ | 24.33 ± 4.48^＊^ |

^*^ P＜0.05; ^**^P＜0.005; ^***^ P＜0.0005

**Table S2** The percentage of apoptotic cell

| **Time** | **PEE** | **Early apoptosis (%)** | **Late apoptosis (%)** |
| --- | --- | --- | --- |
|  | **control** | 2.18 ± 2.03 | 5.50 ± 2.43 |
| **24 h** | **50μg/ ml** | 3.16 ± 1.08 | 8.68 ± 1.15 |
|  | **100μg/ ml** | 4.30 ± 1.78 | 16.89 ± 4.87^＊^ |
|  | **control** | 2.37 ± 2.19 | 4.82 ± 3.22 |
| **48 h** | **50μg/ ml** | 4.80 ± 1.35 | 9.63 ± 1.69 |
|  | **100μg/ ml** | 5.42 ± 1.68 | 13.73 ± 1.55^＊^ |
|  | **control** | 3.84 ± 2.11 | 5.63 ± 0.78 |
| **72 h** | **50μg/ ml** | 7.44 ± 3.09 | 17.08 ± 3.68^＊＊^ |
|  | **100μg/ ml** | 10.08 ± 3.93 | 17.93 ± 1.34^＊＊＊^ |

^*^ P＜0.05; ^**^P＜0.005; ^***^ P＜0.0005
