# Supplementary figures and images for: Suppression of urinary bladder urothelial carcinoma cell by the ethanol extract of pomegranate fruit through cell cycle arrest and apoptosis
Source: BMC Complement Altern Med. 2013 Dec 21;13:364. doi: 10.1186/1472-6882-13-364 (PMC3878077; doi:10.1186/1472-6882-13-364)

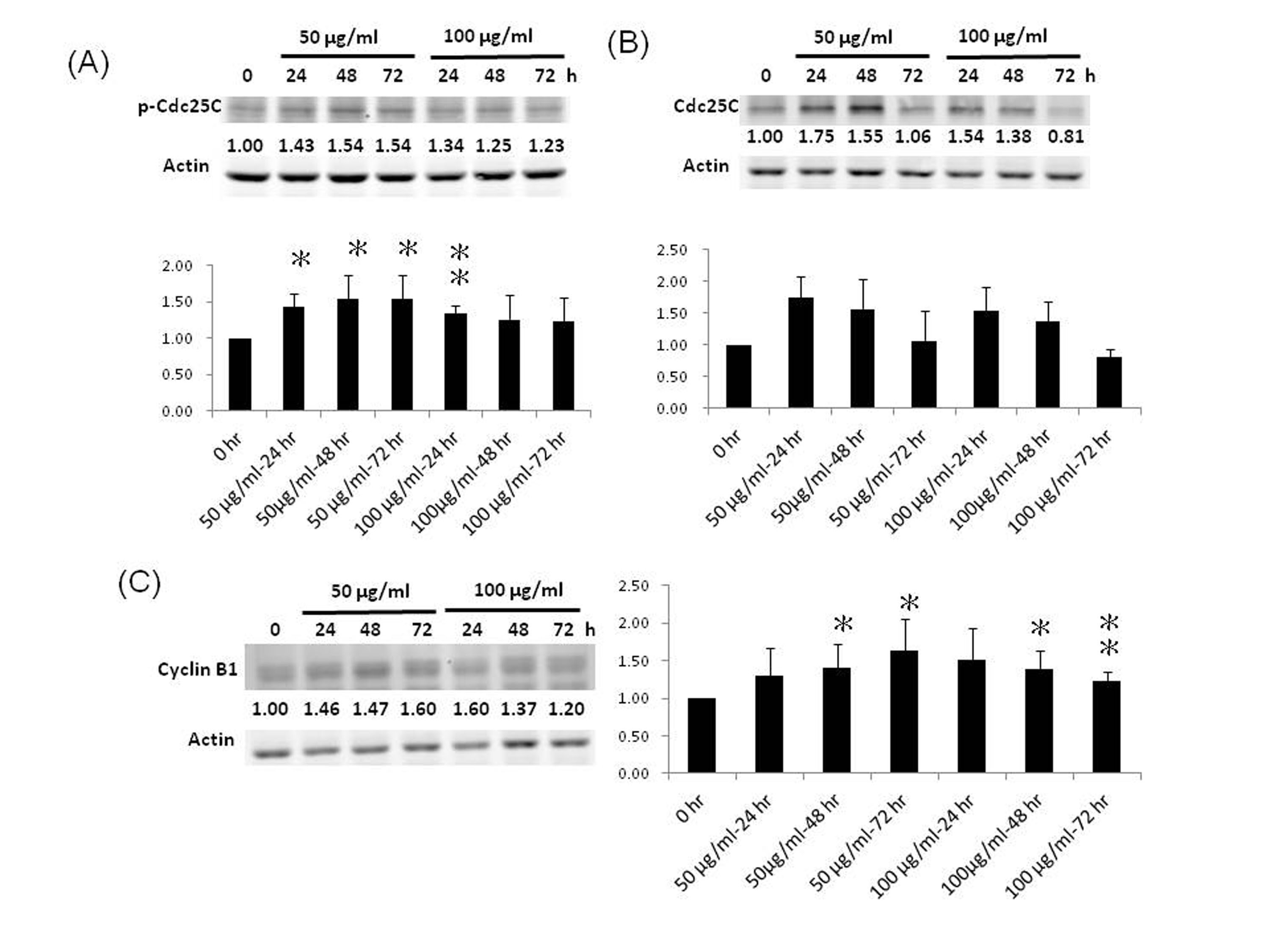

Supplement: Additional file 2 — Supplementary figures. [file 1472-6882-13-364-S2.jpeg]
